# Crystal structure of Usutu virus envelope protein in the pre-fusion state

**DOI:** 10.1186/s12985-018-1092-6

**Published:** 2018-11-26

**Authors:** Zimin Chen, Fei Ye, Sheng Lin, Fanli Yang, Yanwei Cheng, Yu Cao, Zhujun Chen, Guangwen Lu

**Affiliations:** 1 West China Hospital Emergency Department (WCHED), State Key Laboratory of Biotherapy, West China Hospital, Sichuan University, Chengdu, 610041 Sichuan China; 2 Disaster Medicine Center, Sichuan University, Chengdu, 610041 Sichuan China

**Keywords:** Usutu virus, Envelope, Crystal structure, Pre-fusion state, Domain-angle difference

## Abstract

**Background:**

Usutu virus (USUV) is a mosquito-born flavivirus that can infect multiple avian and mammalian species. The viral surface envelope (E) protein functions to initiate the viral infection by recognizing cellular receptors and mediating the subsequent membrane fusion, and is therefore a key virulence factor involved in the pathogenesis of USUV. The structural features of USUV-E, however, remains un-investigated thus far.

**Findings:**

Using the crystallographic method, we determined the structure of USUV-E in the pre-fusion state at 2.0 angstrom. As expected, the overall fold of USUV-E, with three β-barrel domains (DI, DII, and DIII), resembles those of other flaviviral E proteins. In comparison to other pre-fusion E structures, however, USUV-E exhibits an apparently enlarged inter-domain angle between DI and DII, leading to a more extended conformation. Using our structure and other reported pre-fusion E structures, the DI-DII domain-angle difference was analyzed in a pairwise manner. The result shows a much higher degree of variations for USUV-E, indicating the potential for remarkable DI-DII domain angle plasticity among flaviviruses.

**Conclusion:**

We report the crystal structure of USUV-E and show that its pre-fusion structure has an enlarged DI-DII domain-angle which has not been observed in other reported flaviviral E-structures.

## Introduction

The mosquito-born Usutu virus (USUV) was first identified in 1959 in South Africa [2, 32, 53]. Phylogenetically, the virus is closely related to Japanese encephalitis virus (JEV), West Nile virus (WNV) and Murray Valley encephalitis virus (MVEV), and is therefore categorized into the JEV serocomplex within the *Flavivirus* genus of the *Flaviviridae* family [9, 23, 54]. The natural life cycle of USUV involves circulation of the virus between mosquitos and birds such that mosquitos act as vectors and birds as amplifying hosts [8, 45, 48, 49]. In addition to avian species, the virus can also, in incidental cases, be transmitted via mosquito bite to other species including horses, rodents and even humans [5, 10, 46]. Although human cases of USUV infection remain largely asymptomatic, the USUV-related severe diseases such as fever, rash, and meningoencephalitis have also been reported [6, 17, 42]. Furthermore, in laboratory infected mice, the virus could be detected in multiple organs and tissues, including brain, heart, liver, kidney, lung, and etc., demonstrating the pathogenic potential of the virus in mammals [1, 33]. It is notable that several serological surveillance studies have demonstrated the low-prevalence circulation of USUV in the European population, raising the risk of potential USUV outbreaks in humans [19].

As with other flaviviruses, USUV is an enveloped virus which contains a positive-sense, single-stranded RNA genome. This genome encodes a large polyprotein precursor that would be later, via virus-encoded and host proteases, proteolytically processed into three structural (including core (C), pre-membrane (prM), and envelope (E)) and eight non-structural (NS1, NS2A, NS2B, NS3, NS4A, 2 K, NS4B, and NS5) proteins [3]. Of these, the surface-located E protein recognizes cellular receptor/(s) and mediates subsequent fusion between the virus envelope and the lipid bilayer of host cells, and is therefore a key player initiating the viral infection [42]. Structural investigations on flavivirus E protein have revealed a strand-dominated fold with three β-barrel domains [13]. Domain I (DI) is centrally located in the molecule. It connects on one side to domain II (DII) via four polypeptide chains and on the other to domain III (DIII) with a single polypeptide linker. Unlike DI which is overall of globular shape, DII is an elongated structure. A highly hydrophobic fusion peptide is located in this domain, residing at its distal end. DIII folds into an immunoglobulin (Ig)-like structure, and is believed to be involved in receptor binding and also the major target of neutralizing antibodies. In mature virions, E protein assembles, in a “head-to-tail” mode, into pre-fusion dimers, shedding the fusion peptide from premature exposure [27]. During viral entry, E protein would experience large acidic-pH-induced structural re-arrangements via motions of the DI-DII and DI-DIII domain hinges, which would finally lead to the formation of post-fusion trimers and the simultaneous exposure of its fusion peptide for membrane fusion [7]. The functional importance of E in the flaviviral life cycle makes the protein a favorite target for structural studies. While the E structures of multiple flaviviruses, including dengue virus (DENV), JEV, WNV, Zika virus (ZIKV), Tick-born encephalitis virus (TBEV), and etc., have been reported [4, 11, 13, 20, 26–29, 34, 39–41, 51, 57], the structural features of USUV E protein (USUV-E) remains elusive.

In this study, we reported the atomic crystal structure of USUV-E in its pre-fusion state. As expected, USUV-E also folds into three β-barrel domains and exhibits an overall extended conformation. Despite that only a single USUV-E molecule is present in the crystallographic asymmetric unit, a head-to-tail dimer similar to other flaviviral E structures could be observed via symmetry operations. While the overall USUV-E structure resembles those of other flaviviral E proteins, its DII connects to DI in a more extended manner, leading to an apparently enlarged DI-DII inter-domain angle. Via structural comparisons, we further showed that USUV-E, among representative pre-fusion E structures, exhibits a much higher degree of domain-angle variations between DI and DII, which we believe represents potential evidence for a remarkable DI-DII angle-plasticity among flaviviruses.

## Materials and methods

### Gene construction

The coding region for ectodomain residues 1–409 of the envelope protein from USUV (GeneBank: YP_164819) was synthesized by the GENEWIZ corporation and cloned into the bacterial expression vector pET21a with a C-terminal 6 × His-tag by restriction digestion sites of Nde I and XhoI.

### Protein expression and inclusion body preparation

For protein expression, the recombinant plasmid was transformed into *E. coli* BL21 (DE3) cells, and a single colony was picked up and inoculated into 10 ml LB medium for overnight growth. The subsequent cell culture were then transformed into 1 L of fresh LB medium at the volume ratio of 1:100 and allowed to grow at 37 °C until the OD_600_ reached 0.6–0.8. Then, isopropylthiogalactoside (IPTG) was added into the culture at 1 mM to induce the protein expression. After induction at 37 °C for 6 h, the cells were collected and analyzed by SDS-PAGE.

For inclusion bodies preparation, the harvested cells were lysed by sonication in a buffer consisting of 20 mM Tris-HCl, pH 8.0, and 150 mM NaCl. Next, the lysate was centrifuged at 15,000 g, and the pellet containing the protein inclusion bodies was washed three times with 50 ml of wash buffer (50 mM Tris-HCl, pH 8.0, 0.5% Triton X-100, 300 mM NaCl, 10 mM EDTA, 10 mM DTT). Then, the purified inclusion bodies were re-suspended in 40 ml of re-suspension buffer (50 mM Tris-HCl, pH 8.0, 100 mM NaCl, 10 mM EDTA, 10 mM DTT) to remove the residual Triton X-100. After centrifugation at 15,000 g, the final inclusion-body pellet was solubilized in dissolution buffer (6 M Gua-HCl, 10% glycerol, 50 mM Tris-HCl, pH 8.0, 100 mM NaCl, 10 mM EDTA, 10 mM DTT) at 30 mg/ml.

### Protein refolding and purification

The USUV-E protein was expressed as inclusion bodies and then refolded in vitro using the diluted refolding method [25] with some modifications. Briefly, aliquots of inclusion bodies were diluted dropwise into a refolding buffer consisting of 100 mM Tris-HCl, pH 8.0, 600 mM L-Arg HCl, 2 mM EDTA, 5 mM reduced glutathione, 0.5 mM oxidized glutathione, and 10% glycerol, and then refolded overnightat 4 °C. Subsequently, the refolded protein was concentrated using an Amicon Stirred Cell (UFSC40001) concentrator with 10 kDa cut off membrane and then adjusted to 20 mM Tris-HCl, pH 8.0, 150 mM NaCl, and 5% glycerol. Then the refolded E protein was further purified in an AKTA Pure System by gel filtration on a Superdex 200 increase 10/300 GL column (GE Healthcare).

### Crystallization, data collection, and structure determination

The purified USUV-E protein was concentrated to 8 mg/ml. The crystallization trials were performed with 1 μl protein mixing with 1 μl reservoir solution and then equilibrating against 70 μl reservoir solution by sitting drop vapor diffusion at 18 °C. The initial crystallization screening was performed using the commercially-available kits (Hampton Research and Molecular Dimensions). Conditions that can support crystal growth were then optimized with the hanging-drop vapor-diffusion method. High-quality crystals were finally obtained under a condition composed of 0.1 M HEPES, pH 7.5, 10% *w*/*v* Polyethylene glycol 8000, and 8% *v*/v Ethylene glycol. Diffraction data were collected at Shanghai Synchrotron Radiation Facility (SSRF) BL18U1. For data collection, all crystals were cryo-protected by briefly soaking in reservoir solution supplemented with 20% (*v*/v) glycerol before flash-cooling in liquid nitrogen. The collected data were processed using HKL2000 [36].

The structure of USUV-E was determined by the molecular replacement method using Phaser [16] with the structure of WNV-E protein (PDB: 2hg0) as the search model. The atomic model was completed with Coot [14, 15]. Rounds of refinement was performed with REFMAC5 [31], and finally with Phenix[47, 58]. The final model was assessed with PROCHECK [24]. Final statistics for data collection and structure refinement are presented in Table 1. All structural figures were generated using PyMOL (http://pymol.sourceforge.net). The USUV-E structure has been deposited into the Protein Data Bank with a PDB code of 6A0P.

**Table 1:** Data collection and refinement statistics

Data collection	
Space group	P 2_1_2_1_2_1_
Cell dimensions	
*a, b, c* (Å)	35.04,104.34,115.58
α, β, γ (°)	90°,90°,90°
Wavelength (Å)	0.9793
Resolution (Å)^a^	50.0–2.0 (2.07–2.00)
Rmerge^a,b^	0.13 (0.808)
I/σI^a^	13.51 (2.73)
Completeness (%)^a^	99.6 (98.80)
Redundancy^a^	5.5 (4.9)
Total reflections	161,936
Unique reflections	29,488
Refinement	
Resolution (Å)	30.99–2.0
Rwork/Rfree^c^	0.214/0.247
No. of atoms	
Protein	3111
Water	232
B-factors	
Protein	39
Water	42.5
r.m.s.d.	
Bond lengths (Å)	0.018
Bond angles (°)	1.64
Ramachandran plot^d^	
Ramachandran favored (%)	98%
Ramachandran allowed (%)	2%
Ramachandran outliers (%)	0

^a^Values for the outmost resolution shell are given in parentheses

^b^Rmerge = ΣiΣhkl | Ii-<I > | /ΣiΣhklIi, where Ii is the observed intensity and < I > is the average intensity from multiple measurements

^c^Rwork = Σ | | Fo |- | Fc | | /Σ | Fo |, where Fo and Fc are the structure-factor amplitudes from the data and the model, respectively. Rfree is the R factor for a subset (5%) of reflections that was selected prior to refinement calculations and was not included in the refinement

^d^Ramachandran plots were generated by using the program MolProbity

## Results and discussion

**Fig. 1:**
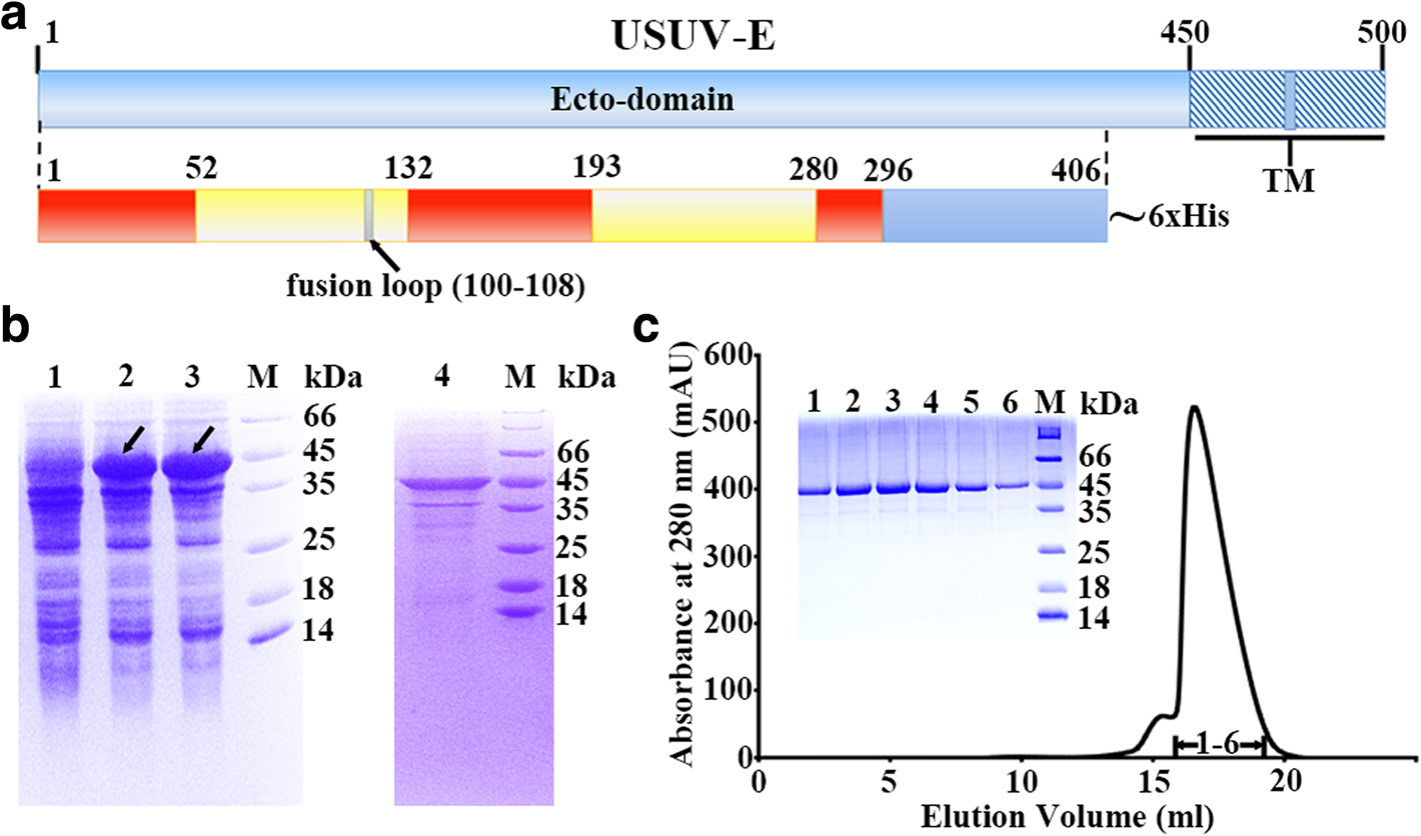
Expression and purification of the USUV-E protein. **a** A schematic view of USUV-E. The ecto-domain and the transmembrane domains (TM) of the protein are indicated. For USUV-E preparation, its ecto-domain region spanning residues 1–406 was expressed as a fusion protein with a C-terminal 6xHis tag. The three domains of this ecto-domain protein are highlighted in red, yellow, and blue, respectively, and the fusion loop is in grey. **b** Small-scale expression and inclusion-body extraction. The SDS-PAGE results are shown. Lane 1, un-induced; lane 2, induction with 0.2 mM IPTG; lane 3, induction with 1 mM IPTG; lane 4, the extracted inclusion bodies. The USUV-E protein was marked with arrows. **c** Purification of the refolded USUV-E protein by size exclusion chromatography. The separation chromatograph of the protein and the SDS-PAGE analyses of the pooled samples are shown

USUV-E, with a canonical type I transmembrane topology, contains a large N-terminal ecto-domain (residues F1-R450) (Fig. 1a). To gain insight into the structural features of USUV-E, the ecto-domain region of the protein spanning amino acids F1-A406 was first engineered to include a C-terminal 6xHis tag and then expressed in *E. coli*. Following several previous reports on flaviviral E-preparations [13, 20, 27], the resultant USUV-E protein was initially expressed in the form of inclusion bodies (Fig. 1b), and subsequently refolded and further purified to homogeneity by gel filtration chromatography (Fig. 1c).

Via crystallization screening, crystals of USUV-E that can diffract to 2.0 Å were successfully obtained via the hanging-drop vapor-diffusion method. The structure was solved by molecular replacement and finally refined to Rwork = 0.214 and Rfree = 0.247, respectively (Table 1). Within the crystallographic asymmetric unit, a single USUV-E molecule was present, and clear electron densities were successfully traced for USUV-E amino acids F1-R406 as well as for four terminal His residues of the fusion tag.

**Fig. 2:**
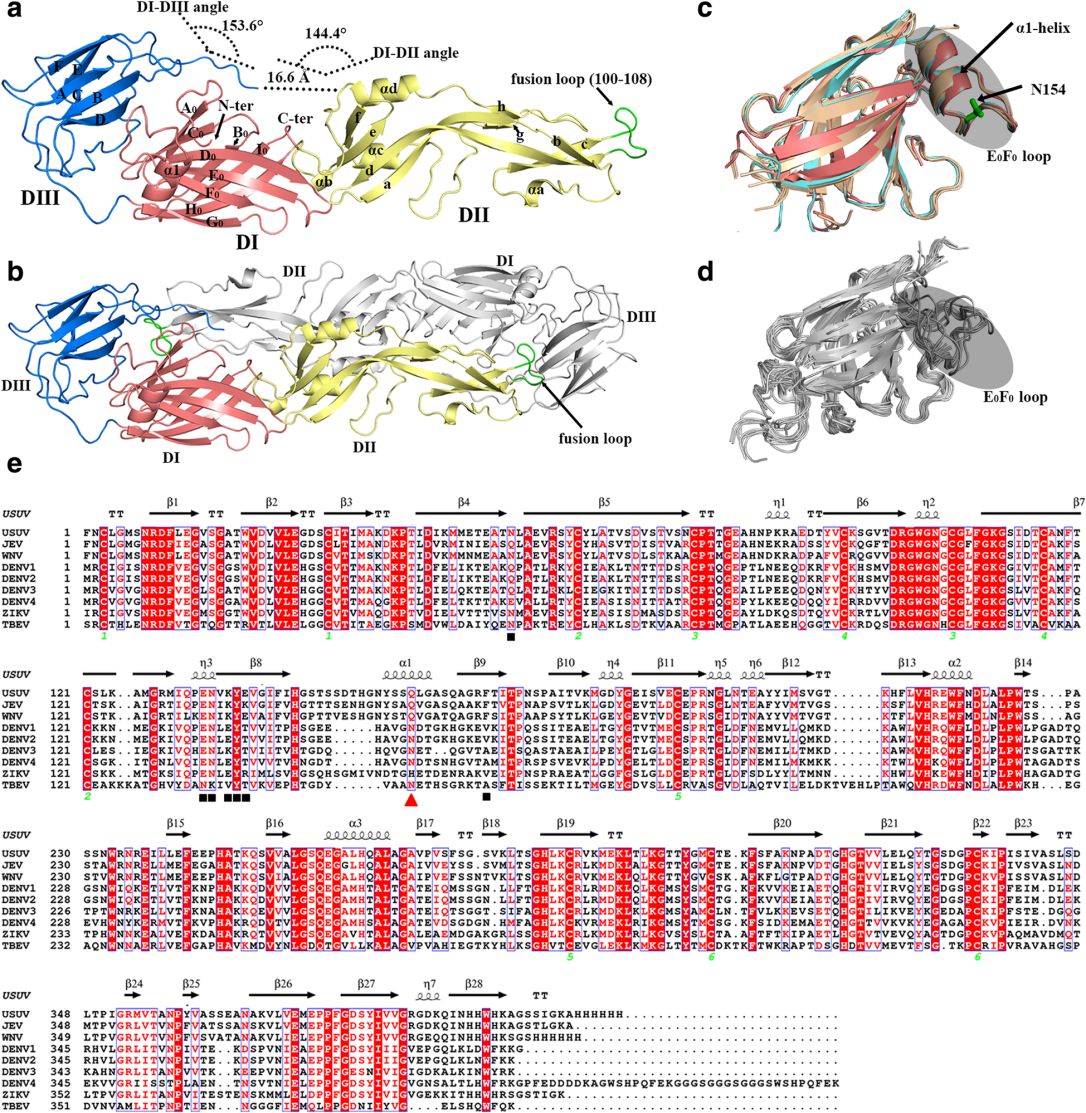
Overall Structure of the USUV-E protein. **a** An overview of the solved structure. The three domains (DI, DII, and DIII) are colored in red, yellow, and blue, respectively, and the fusion loop is in green. The inter-domain angles between DI and DII and between DI and DIII, which are calculated to be 144.4° and 153.6°, respectively, are highlighted. All the secondary structure elements (followed the nomenclature reported for ZIKV envelope [13]) referred to in the text are labeled. **b** A head-to-tail USUV-E dimer generated by symmetry operations. The original USUV-E molecule is colored as in panel a, and the symmetry related molecule is in grey. The buried fusion loop is highlighted in green. **c** Superimposition of the DI of E-structures of JEV serocomplex (E-USUV marked in red ribbon, E-WNV in cyans, and E-JEV in wheat tint); highlighting their E_0_F_0_ loops (shaded for clarity) and the loop-located α1 helices. **d** Superimposition of the DI of E-structures of other flaviviruses (the JEV serocomplex members excluded). Clearly shown is that the α1 helix is not present in these structures, and the E_0_F_0_ loop is of variable conformation. **e** Structure-based multiple sequence alignment of representative flaviviral E proteins. Horizontal arrows indicate β-strands and spinal lines highlight α-helices. The Asn residue that could be glycosylated in the E_0_F_0_ loop is marked with a red triangle, and those residues recognized by CR4354 are highlighted with black boxes

As expected, USUV-E also folds into three domains (DI, DII, and DIII), showing an extended and a β-dominated structure (Fig. 2a). DI is composed of nine β-strands (A_0_-I_0_), forming a compact barrel structure. One helix (α1) is present in DI, sterically situating in the vicinity of the A_0_C_0_D_0_E_0_F_0_ sheet and also of the molecule N-terminus (Fig. 2a). It is notable that this helix is also observed in the structures of E proteins from other JEV serocomplex members (eg. WNV and JEV) but not of other flaviviral E proteins (Fig. 2c-d). We also noted that this helix is, along the E protein sequence (Fig. 2e), located in the E_0_F_0_ inter-strand loop (also called the glycan loop) where a glycosylation site locates. The USUV-E protein prepared in this study, however, lacks any glycosylation modifications at this site due to expression in *E. coli*. Nevertheless, when comparing our protein to a similarly eubacteria-yielded JEV protein without glycan decoration and an insect-cell-generated WNV protein that contains glycan modifications, their structures revealed both well-aligned E_0_F_0_ loops and the α1 helices (Fig. 2c). The formation of such a helix in the USUV, JEV, and WNV E proteins is therefore unlikely affected by the glycosylation status of this glycan loop. In light of the high conformation-variability observed for this glycan loop in other flaviviral E structures (Fig. 2d), we believe this extra α1 helix is likely a novel feature of the JEV serocomplex viruses. Sterically, DI is located in the center of the E molecule and is further flanked on one side by DII and on the other by DIII (Fig. 2a). DII consists of 8 strands (a-h) and 4 helices (αa-αd), exhibiting a rather extended conformation. This domain can be further divided into two subdomains (subdomain I and II). The latter directly connects to DI via four polypeptide linkers and is composed of a four-stranded (a, d, e, and f) anti-parallel β-sheet and three α-helices (αb, αc, αd); while the former is located at the distal end of the molecule, showing a β-barrel fold with three long strands (b, c, and d) at the bottom and two small ones (g and h) on the top. The fusion peptide, with amino acids G100-F108 is residing in subdomain I. It is also noteworthy that the DII d strand, which is of great length, exhibits a twisted conformation and extends from subdomain I to subdomain II (Fig. 2a). DIII is also a β-barrel structure. It contains in total 6 anti-parallel β-strands (A-F), assembling into an overall Ig-like fold. The inter-domain angles between DI-DII and DI-DIII were calculated to be 144.4° and 153.6°, respectively. On the whole, the solved USUV-E structure is quite similar to those of other flaviviral E proteins [13, 20, 27, 29, 34, 39, 41].

We then characterized the possible dimeric architecture of USUV-E using our structure. As expected, a head-to-tail USUV-E dimer which resembles previously reported flaviviral pre-fusion E-dimers could be generated via simple symmetry operations (Fig. 2b). In this dimer form, the highly hydrophobic fusion loop is readily concealed from the bulk solvent by the DIII domain of the other molecule.

**Fig. 3:**
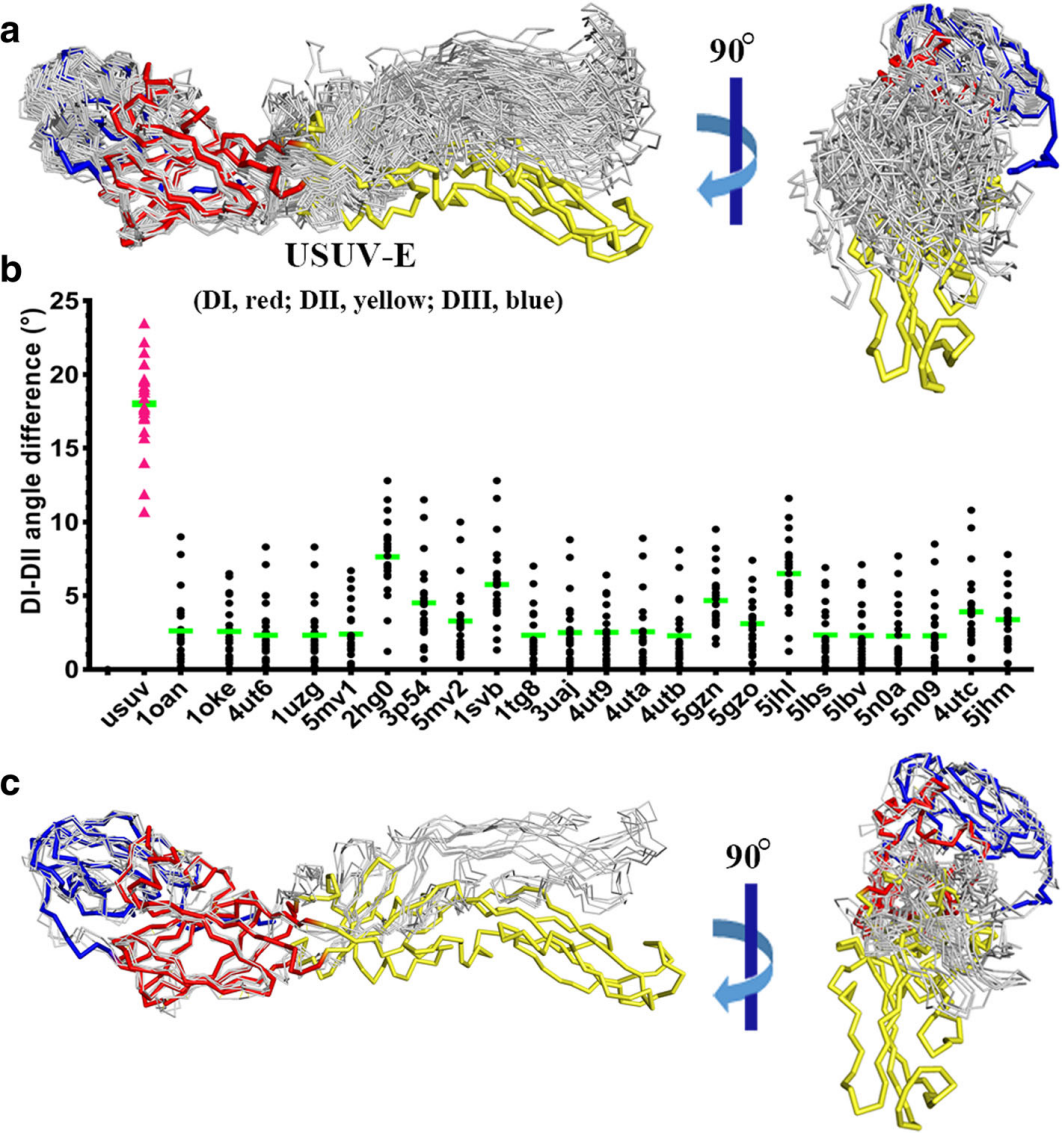
Comparison of USUV-E structure with other reported crystal and cryo-EM pre-fusion flaviviral E-structures. **a** Superimposition of the crystal E-structures. The pre-fusion E-structures selected for comparison include those from DENV2 (PDB code: 1tg8, 1oan, 1oke, 4ut6, 4ut9, 4uta, 4utb, and 4utc), DENV3 (PDB code: 1uzg), DENV4 (PDB code: 3uaj), WNV (PDB code: 2hg0), JEV (PDB code: 3p54, 5mv1, 5mv2), TBEV (PDB code: 1svb), and ZIKV (PDB code: 5gzn, 5gzo, 5jhl, 5jhm, 5lbs, 5lbv, 5n0a, and 5n09). The USUV-E structure is colored red for DI, yellow for DII, and blue for DIII, respectively, and the rest E-structures are colored grey. The right panel is yielded by rotation of the structure for about 90° around a vertical axis. Clearly shown is that while the DI and DIII domains could be well aligned, DII connects to DI in a more extended manner in USUV-E, resulting in an obviously enlarged inter-domain angle. **b** A quantitative comparison of the DI-DII domain-angle variations characterized in panel a. For each structure, its DI-DII domain-angle was compared to those of other structures, and the angle-difference was calculated for each structure pairs and then plotted as black dots. The angle-difference of USUV-E (relative to other flaviviral E proteins) was marked with red triangle. The mean values was represented with horizontal green lines. **c** Superimposition of our structure with representative cryo-EM pre-fusion E-structures including those from JEV (PDB code: 5ywo, 5wsn), ZIKV (PDB code: 6co8), and DENV-2 (PDB code: 3J27). The molecules are oriented and colored the same as in panel a

To further characterize the similarities and differences between our structure and other reported pre-fusion E crystal structures, those from DENV [28, 41, 57], JEV [26, 27], WNV [34], ZIKV [4, 13, 28, 51], and TBEV [39] (either in the free form or in the antibody-bound form) were selected and superimposed for structural comparison. While the DI and DIII domains could be well aligned, an astonishing oriental difference was observed for the DII domain such that it connects to DI in a more extended manner in USUV-E and therefore leads to an obviously enlarged inter-domain angle (Fig. 3a). This makes USUV-E an E protein with the largest DI-DII angle characterized thus far. To learn, in a more quantitative way, the observed domain-angle variations between DI and DII among these structures, the angle-difference was calculated for each structure pairs and then plotted in cluster for each structure group. As shown in Fig. 3b, in comparison to other E-structures which yield angle-differences ranging from ~ 0–12.8° (with an average ranging from ~ 3–7.8°), a much higher degree of variations was observed for USUV-E (with angle-differences ranging from ~ 10.7–23.5° and an average of ~ 18°). It is notable that such domain-angle variations between DI and DII have been widely observed in crystal structures of flaviviral E proteins, for which the crystal packing forces might also play a role. We therefore selected the cryo-EM structures of JEV [38, 52], ZIKV [43], and a representative dengue virus (DENV-2) [56] for a similar domain-angle comparison. Similar to those observed with the E crystal structures, USUV-E is also the most extended pre-fusion structure analyzed in this study (Fig. 3c). We also noted that four His residues of the fusion tag are also traceable in our structure and extend towards DII. Nevertheless, these amino acids are far away (~ 16.6 Å) (Fig. 2a) from physically interacting with DII residues and are unlikely the reason causing the increased DI-DII angle. We therefore believe the enlarged domain-angle between DI and DII in USUV-E is an intrinsic structural feature of this viral protein. It should be noted that the inter-domain motion is commonly observed in flaviviral E proteins and has been proposed as a prerequisite for the pre-fusion to post-fusion transitions [37, 44]. Nevertheless, the DI-DII angle-difference as high as shown for the USUV-E structure reported in this study has not been observed previously. We believe this may represent potential evidence for a remarkable DI-DII domain-angle plasticity among flaviviruses. Our structure also indicates a notion that flaviviruses have evolved to adjust to accommodate species-specific dimeric arrangements featured with their variant DI-DII domain-angles.

Finally, a couple of potent neutralizing monoclonal antibodies (mAbs) against JEV and WNV, including A3, B2, E3, E113, NARMA3, 503, and CR4354, have been reported [12, 18, 22, 30, 35, 50, 55]. It is notable that among these, CR4354 recognizes E-epitopes locating in the DI-DII hinge region [21, 50]. We noticed that the hinge-residues recognized by CR4354 are largely preserved in USUV-E (Fig. 2e). Nevertheless, the observed enlarged DI-DII domain-angle of the USUV protein raises the possibility that these amino acids might occupy variant steric locations. It is therefore worth studying in the future to learn if CR4354 (or any other JEV serocomplex mAbs that target the DI-DII hinge region) could also be potentially used to prevent USUV infections.

## Abbreviations

DENV: Dengue virus
DI/DII/DIII: DomainI/DomainII/DomainIII
E: Envelope
IPTG: Isopropylthiogalactoside
JEV: Japanese encephalitis virus
MVEV: Murray Valley encephalitis virus
prM: pre-Membrane
SSRF: Shanghai Synchrotron Radiation Facility
TBEV: Tick-born encephalitis virus
USUV: Usutu virus
WNV: West Nile virus
ZIKV: Zika virus
